# Treatment of Erythematotelangiectatic Rosacea With Collateral Puncture Therapy: Protocol for a Randomized Controlled Trial

**DOI:** 10.2196/59682

**Published:** 2025-06-17

**Authors:** Aolin Song, Bingnan Cui, Xuemin Wang, Jianing Bi, Xiaohong Wu, Liu Yang, Li Liu, Shengyuan Qu, Zhu Fan, Jiao Yang, Yuhe Yan

**Affiliations:** 1 Department of Dermatology Guang’anmen Hospital Beijing China; 2 Traditional Chinese Medicine Department Shanghai Skin Disease Hospital Shanghai China

**Keywords:** erythematotelangiectatic rosacea, collateral puncture, intense pulsed light, randomized controlled trial, protocol

## Abstract

**Background:**

Erythematotelangiectatic rosacea (ETR) is the most common subtype of rosacea, characterized by persistent facial erythema and telangiectasia of varying calibers. It causes significant aesthetic impairment and is often accompanied by uncomfortable symptoms, such as burning, stinging, dryness, and itching, profoundly affecting patients’ quality of life. Intense pulsed light (IPL) therapy demonstrates notable improvement in persistent erythema and telangiectasia; however, it is associated with issues such as a prolonged treatment course and high costs. Collateral puncture therapy involves rapid puncturing of specific acupuncture points followed by gentle squeezing around the needle holes to induce minor bleeding. Previous studies have shown that collateral puncture therapy for ETR offers advantages such as rapid onset of effect, a simple procedure, and low cost. Nevertheless, more high-quality clinical research data are needed to confirm these findings.

**Objective:**

This study aims to observe the clinical efficacy and safety of collateral puncture therapy in treating ETR.

**Methods:**

This study enrolled 60 patients diagnosed with ETR. The patients were randomly divided into 2 groups: one group underwent 4 sessions of collateral puncture therapy with 1-week intervals between treatments, and the other group received a single session of IPL therapy. The primary efficacy end points were the clinician’s erythema assessment and the clinician’s telangiectasia assessment. The secondary efficacy end points included the investigator’s global assessment, patient’s self-assessment, Flushing Assessment Tool results, Dermatology Life Quality Index, and Rosacea-specific Quality-of-Life instrument. The evaluation points were before treatment, immediately after treatment, and during follow-up. The data were statistically analyzed using SPSS (version 25.0; IBM Corp) to compare intragroup and intergroup differences between the 2 sets of data before and after treatment, with a significance level of α=.05 for hypothesis testing.

**Results:**

Recruitment began on June 1, 2023. All participants have been recruited. Data analysis will be complete by the end of August 2025, with study findings available by December 2025.

**Conclusions:**

This study has the potential to verify the clinical efficacy and safety of collateral puncture therapy in the treatment of ETR, supplement rosacea treatment methods, standardize treatment protocols, and fill a current clinical gap in treating rosacea.

**Trial Registration:**

Chinese Clinical Trial Registry ChiCTR2200062639; https://www.chictr.org.cn/showproj.html?proj=177100

**International Registered Report Identifier (IRRID):**

DERR1-10.2196/59682

## Introduction

### Background

Rosacea initially presents with facial flushing, papules, and pustules, which, if recurrent, develop into persistent erythema and varying sizes of telangiectasia [1-3]. A 2019 survey of 10,095 residents in Changsha, China, showed a local prevalence of rosacea of 3.48% [4]. A 2020 epidemiological survey among 9227 university students from 2 universities showed a prevalence of 3.4% [5]. Facial appearance and features significantly impact a person’s professional, social, and romantic life [6]. The common perception of facial redness is that it signifies anger, embarrassment, or excessive drinking, and this could potentially cause shame in patients with rosacea [7], leading to various sociopsychological impacts and psychological complications, such as social anxiety, shame, and depression; hence, there is a strong desire for treatment among these patients.

Current clinical treatments for erythematotelangiectatic rosacea (ETR) mainly involve topical and oral vascular regulators and photophysical therapy. Topical treatments with 0.5% brimonidine tartrate gel and 1% oxymetazoline hydrochloride cream [8], which are α-adrenergic receptor agonists, are commonly used. These medications specifically act on the smooth muscles around facial blood vessels, constricting vessels and reducing persistent central facial erythema, but are ineffective on dilated capillaries, papules, or pustules. It is believed that improvement in erythema is only a temporary suppression. The main oral treatment is the β-adrenergic receptor inhibitor carvedilol [8]. Carvedilol has both α1 receptor blocking and nonselective β receptor blocking effects, acting on myocardial β1-adrenergic receptors to slow the heart rate and ease patient tension; this is mainly used for refractory transient flushing and prominent persistent erythema. Despite good patient tolerance, caution is advised for hypotension and bradycardia, with monitoring of heart rate and blood pressure recommended. Some patients experience rebound upon discontinuation. In photophysical therapy, options include intense pulsed light (IPL), pulsed dye lasers, potassium titanyl phosphate lasers, Nd: YAG lasers, and radiofrequency therapy [9]. Laser devices use hemoglobin as a medium, emitting photons or laser beams to denature and coagulate hemoglobin, forming blood clots and damaging endothelial cells within capillaries, reducing telangiectasia and inhibiting vascular proliferation. While laser therapy has advantages for treating telangiectasia, patients with severely compromised skin barriers cannot tolerate the local stimulation of lasers, and the high cost of laser treatment limits its widespread clinical use.

Collateral puncture therapy is one of the oldest healing methods in human medical history, dating back to the Stone Age, when it was discovered that collateral puncture could alleviate pain [10], leading to the development of medical tools like *bian* stones. With the progress of history, various acupuncture and collateral puncture tools were developed [11], including acupuncture needles and triangular needles. Collateral puncture therapy involves using needles to puncture superficial small veins, specific acupoints, lesion areas, or pathological reaction points to release a certain amount of blood for treating diseases [12]. “Meridians” in *Miraculous Pivot*, a classical work in Chinese medical literature, mentions that the main function of collateral puncture therapy is dredging channels and regulating *qi* and blood for therapeutic effects. In traditional Chinese medicine, the pathogenesis of rosacea is the stagnation of “heat evil” in the facial skin, causing blood coagulation, which aligns well with the disease and makes collateral puncture therapy suitable for its treatment. Additionally, modern medicine recognizes that collateral puncture can improve microcirculation, making it particularly suitable for treating ETR [13].

Clinically, collateral puncture therapy has been widely applied in the treatment of rosacea and has received positive feedback on its efficacy [14-18]. However, there is currently a lack of high-quality medical evidence to support it. Therefore, this project aims to objectively evaluate the efficacy of collateral puncture therapy on ETR using scientific clinical observation methods.

### Objectives

The primary objective is to verify the clinical efficacy and safety of collateral puncture therapy in the treatment of ETR. The secondary objective is to supplement rosacea treatment methods, standardize treatment protocols, and fill a current clinical gap in treating rosacea.

## Methods

The study protocol was designed in accordance with the Standard Protocol Items: Recommendations for Interventional Trials (SPIRIT) guidelines (Multimedia Appendix 1) [19].

### Study Design and Setting

This is a randomized, controlled, evaluator-blinded trial. Sixty patients were evenly divided into the experimental group and the control group.

### Recruitment and Informed Consent

All participants were recruited from Guang’anmen Hospital, China Academy of Chinese Medical Sciences. Public recruitment advertisements for this trial were used to recruit patients both online and offline (eg, through WeChat public accounts and websites). The researchers determined patient eligibility for this study based on strict application of the inclusion and exclusion criteria. Interested and willing patients who met the inclusion criteria signed a written informed consent form (Multimedia Appendix 2) before the start of the study. They were fully informed about the study, including its procedures, benefits, and potential risks. Participants were free to withdraw from the study at any time without penalty and without any negative impact on their future medical care.

### Study Population

#### Inclusion Criteria

Patients were included if they (1) met the diagnostic criteria for rosacea as defined in the “Guidelines for the Diagnosis and Treatment of Rosacea in China (2021 Edition),” [20] specifically the erythematotelangiectatic type; (2) were aged between 18 and 70 years (inclusive); and (3) signed an informed consent form.

#### Exclusion Criteria

Patients were excluded if they (1) had any systemic or active skin disease on the face that could affect the evaluation of the study results (such as connective tissue disease) or had scars, tattoos, birthmarks, or pigmentary skin diseases on the affected area that could affect the assessment of skin lesions; (2) had serious primary diseases of the heart, cerebral vessels, liver, kidney, or hematopoietic system; mental illnesses or a history of autoimmune diseases; were pregnant or breastfeeding; had photosensitivity; or had a fear of needles; (3) used oral immunomodulators (such as hydroxychloroquine sulfate), β-adrenergic receptor inhibitors (such as carvedilol), or antianxiety drugs (such as mirtazapine and paroxetine) for rosacea treatment within 30 days before treatment; (4) had applied vasoconstrictor drugs on the face (such as 0.5% brimonidine tartrate gel or 1% hydroxymetazoline hydrochloride cream) within 7 days before treatment; used calcineurin inhibitors, antimicrobials, or steroid creams; or had undergone fire-needle therapy, collateral puncture, or LED light treatment on the face; (5) had undergone facial injections, laser treatments, or chemical peels within 30 days before treatment; (6) were currently participating in, or had participated in, another clinical trial within the last 3 months; or (7) could not tolerate any of the procedures involved in the trial.

#### Removal Criteria

Patients were removed from the study if they (1) showed poor compliance during the trial, affecting the determination of efficacy and safety; (2) experienced serious adverse events, complications, or special physiological changes, making them unsuitable to continue the trial (these were recorded as adverse reactions); (3) were concomitantly using nonprescribed medications, especially those significantly affecting the trial medication, impacting the determination of efficacy and safety; (4) voluntarily withdrew during the trial; (5) withdrew from the trial before completing the course, were lost to follow-up, or died for any reason; or (6) had incomplete data, affecting the judgment of efficacy and safety.

### Randomization and Blinding

Sixty patients were randomly divided into an experimental group and a control group, with 30 in each, using a random controlled method. To avoid selection bias, random numbers were generated using SAS (SAS Institute). The allocations were sealed in opaque, carbonless copy-paper envelopes. After participants agreed to random allocation, they received an allocation sequence number. Upon opening the corresponding envelope, group assignment was determined and recorded in the case report form by a dedicated person. Throughout the study, the evaluators and statisticians were blinded to group allocation.

### Intervention

#### Instrument Selection

The triangular needles used in the study (Huatuo brand disposable acupuncture needles; Suzhou Medical Supplies Factory; production license number 20010020 of the Su Food and Drug Administration of Machinery Production; registration certificate number 20210456). The needle size was 1.6 mm×65 mm. The IPL therapy device was the Icon MaxG (Cynosure LLC).

#### Collateral Puncture Group

**Figure 1:**
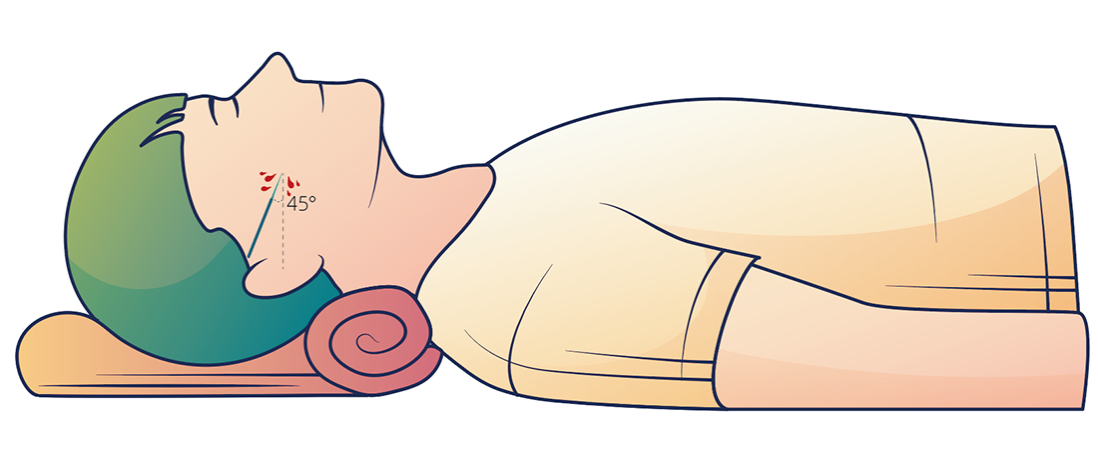
Diagram of collateral puncture therapy.

Patients in the experimental group underwent facial collateral puncture therapy at the dermatology department of Guang’anmen Hospital. The selected acupuncture points were *ashi* points located at the sites of facial telangiectasia. Prior to treatment, the practitioner massaged the chosen areas to induce congestion. During the procedure, a triangular needle was rapidly and shallowly inserted into the skin at an oblique angle to ensure that blood flowed naturally without puncturing blood vessels and causing hematoma. The volume of bloodletting was controlled within the range of 0.5 to 1 ml. The treatment schedule consisted of 1 session of collateral puncture per week for a total of 4 treatments. Throughout the entire trial, collateral puncture procedures were performed by the same acupuncturist (Figure 1).

The term “*ashi* point” originates from Sun Simiao’s *Essential Prescriptions Worth a Thousand Gold*. These points can be found anywhere on the body and represent a phenomenon known as temporary acupuncture points. When a disease arises, there is a corresponding stagnation of *qi* and blood in a specific body part, leading to localized and temporary accumulation of *qi* and blood, thus manifesting the *ashi* point phenomenon. As the disease subsides, the temporary accumulation of *qi* and blood dissipates, and the *ashi* point vanishes. Therefore, according to traditional Chinese medicine, *ashi* points serve as both indicators of disease and the best stimulation point for treatment.

#### IPL Group

Patients in the control group underwent facial IPL treatment in the laser department of Guang’anmen Hospital, specifically targeting the localized facial telangiectasia. The IPL wavelengths used were 500 to 670 nm and 870 to 1200 nm, with a spot size of 10 mm×15 mm, a pulse width of 20 ms, and an energy density ranging from 36 to 38 to 40 J/cm^2^. The overlap of control points was maintained within a 10% range. The treatment protocol consisted of a single session administered upon enrollment, totaling 1 treatment. Throughout the trial, all laser procedures were performed by the same laser physician.

### Outcomes

The study timeline is presented in Table 1. All outcome measures shown in the table were recorded from baseline to the end of follow-up.

**Table 1:** Study schedule for data measurements.

Time point			Study period (days)											
			Baseline		Treatment									Follow-up
			0		0		7±3		14±3		21±3		28±3	
**Enrollment**														
	Sign informed consent	✓												
	Inclusion/exclusion criteria	✓												
	Medical history	✓												
	Demographic data	✓												
**Assessment**														
	Clinician erythema assessment	✓		✓								✓		
	Clinician’s telangiectasia assessment	✓		✓								✓		
	Investigator’s global assessment			✓								✓		
	Patient’s self-assessment			✓								✓		
	Flushing Assessment Tool	✓										✓		
	Dermatology Life Quality Index	✓										✓		
	Rosacea-specific Quality-of-Life	✓										✓		
	Adverse reactions	✓		✓		✓		✓		✓		✓		
	Combination medication/treatment	✓		✓		✓		✓		✓		✓		
	Adverse event reporting	✓		✓		✓		✓		✓		✓		
	Research completion status	✓		✓		✓		✓		✓		✓		
	Researcher review	✓		✓		✓		✓		✓		✓		

#### Primary Outcomes

The clinician erythema assessment [21] assesses the severity of erythema before treatment, immediately after treatment, and at follow-up. The clinician’s telangiectasia assessment [22] assesses the severity of capillary dilation, classified into 5 grades based on the number and size of dilated vessels, with evaluations conducted before treatment, immediately after treatment, and during the follow-up period.

#### Secondary Outcomes

The investigator’s global assessment [23] provides an overall assessment of the treatment’s effectiveness immediately after treatment and at follow-up. The patient’s self-assessment [24] assesses the patient’s own perception of the treatment immediately after treatment and at follow-up. The Flushing Assessment Tool [25] assesses facial flushing before treatment and at follow-up. The Dermatology Life Quality Index and Rosacea-specific Quality-of-Life [26,27] tools rate the patient’s quality of life before treatment and at follow-up (Multimedia Appendix 3).

### Sample Size

With reference to relevant literature, we used a noninferiority trial calculation formula for sample size calculation, as follows:

n1=[(u1-T+u1-u)e/W]2×(1+c)/c, n2=cn1 [28]

In the formula, where *c*=1, *n2*=*n1*; setting α=.05, then *u1*–*T*=1.64, *u1*–*u*=1.28. Based on previous studies using clinician erythema assessment scoring as the primary efficacy end point, *e* is taken as 0.84, *W* as 1. Substituting the data into the formula, we calculate *n1*=*n2*≈13 [29]. Considering a 30% dropout rate and to enhance data reliability, the final sample size was set at 30 cases per group and 60 cases in total.

### Data Management and Confidentiality

The research team will meticulously record the initial data for each participant on the case report form and systematically input them into a secure Microsoft Excel database. Participants’ identities will be anonymized and represented by unique random numbers, ensuring the researchers cannot access their personal information. This approach guarantees stringent data confidentiality. The task of data storage and management, as well as rigorous verification of data accuracy, rests with a designated data custodian. The investigator is responsible for diligently completing each participant’s case report form. The research director will periodically audit the data collection process to maintain its integrity. To safeguard participant privacy, all personal information, including names, contact details, and medical records, will be anonymized and securely stored. Such data will be housed in a specially designated cabinet under the researchers’ custody and preserved for a minimum of 5 years after publication. The Ethics Committee of Guang’anmen Hospital, China Academy of Chinese Medical Sciences, will routinely oversee the trial’s progression. This includes monitoring the collection, allocation, and concealment of data and ensuring adherence to ethical standards. The committee holds the authority to propose modifications or terminate the trial if necessary. The Data Monitoring Committee, acting independently from the sponsors and devoid of any conflicts of interest, will provide unbiased oversight of the trial’s data management processes.

### Adverse Event Reporting and Safety Monitoring

During the study, adverse reactions could occur at the lesion site, such as local bruising, erythema, edema, pain, itching, blisters, exudation, dry skin, desquamation, acne-like lesions, skin sensitivity, hyperpigmentation, and scarring. If adverse reactions occurred, we recorded the adverse event; if adverse reactions occurred after treatment, we considered their relation to the treatment, reported them promptly to the researcher, and if necessary, stopped the treatment and provided corresponding symptomatic treatment.

### Ethical Considerations

The protocol study has received ethical clearance from the Ethics Committee of Guang’anmen Hospital, China Academy of Chinese Medical Sciences (2022–127-KY). Patients have the right to withdraw from this study at any time. Prior to enrollment, we ensured that each patient had a clear understanding of the informed consent form, and it was the responsibility of the investigating physician to ensure that every patient provided informed consent before entering the study. The informed consent forms will be retained as part of the clinical study documentation for future reference. We are committed to safeguarding participants’ privacy and confidentiality by anonymizing all data and storing them securely, with access restricted solely to the research team. Although participants did not receive financial compensation, we express our sincere gratitude for their contributions. Dissemination of the study’s findings is planned through publication in a reputable, peer-reviewed academic journal.

## Results

**Figure 2:**
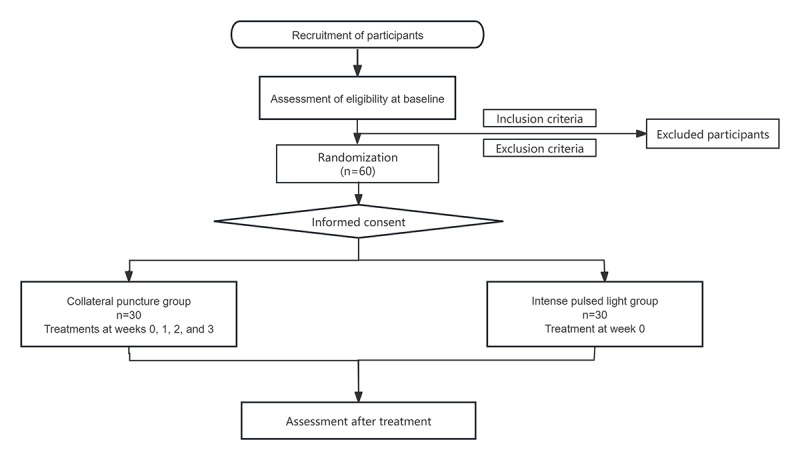
Flowchart of the research procedure.

The research protocol was approved by the Guang’anmen Hospital Ethics Committee on June 24, 2022, and was registered on August 14, 2022. Recruitment commenced on June 1, 2023, with an average of 4 to 6 participants enrolled per month. All patients have been recruited, and we anticipate publishing our research findings by the end of 2025 (Figure 2).

Statistical analysis will be conducted using SPSS (version 25.0; IBM Corp). Means and SDs will be calculated, and measurement data will be analyzed using 1-tailed *t* tests and ANOVAs. Nonparametric tests will be used for data not following a Gaussian distribution; the *χ*^2^ test will be used for categorical data. Based on previous research outcomes, we anticipate that when comparing the primary and secondary efficacy indicators between the follow-up and baseline periods, scores in both the treatment and control groups will show a downward trend. However, we expect that the decline in scores will be more pronounced in the experimental group.

## Discussion

We recruited a total of 60 patients with ETR to conduct a randomized, blinded, controlled study, aiming to observe the therapeutic effect of collateral puncture therapy on ETR patients by comparing the outcomes before and after treatment. Our hypothesis is that, compared to patients receiving IPL therapy, those undergoing collateral puncture will exhibit significant improvements in erythema and telangiectasia. To our knowledge, no studies have been reported that directly compare the pure traditional Chinese medicine technique of collateral puncture with IPL for the treatment of ETR. This study intends to fill the gap in the direct comparison between traditional nonpharmacological therapies in Chinese medicine and modern photoelectronic technologies.

The pathogenesis of rosacea involves contributions from ultraviolet rays, bacteria, demodex folliculiformis, immune dysregulation, neurovascular dysfunction, and genetic factors, among others [20]. Collectively, these etiological agents induce an inflammatory response in the body. IPL therapy for patients with rosacea uses the principle of selective photothermolysis, where the laser specifically targets diseased tissue while sparing adjacent normal tissue, causing thermal damage to dilated capillaries, leading to their coagulation and necrosis [30,31]. Bipolar radiofrequency therapy converts electrical energy into thermal energy through the epidermis to further heat the target tissues. When combined, IPL and bipolar radiofrequency therapy exhibit synergistic effects in reducing dilated capillaries and inflammatory cell infiltration, resulting in favorable therapeutic outcomes. IPL is a commonly used treatment modality for ETR [32]; however, it is relatively costly and has limited efficacy in alleviating symptoms such as facial warmth and itching. Therefore, it was selected as the intervention for the positive control group in this study [33].

**Figure 3:**
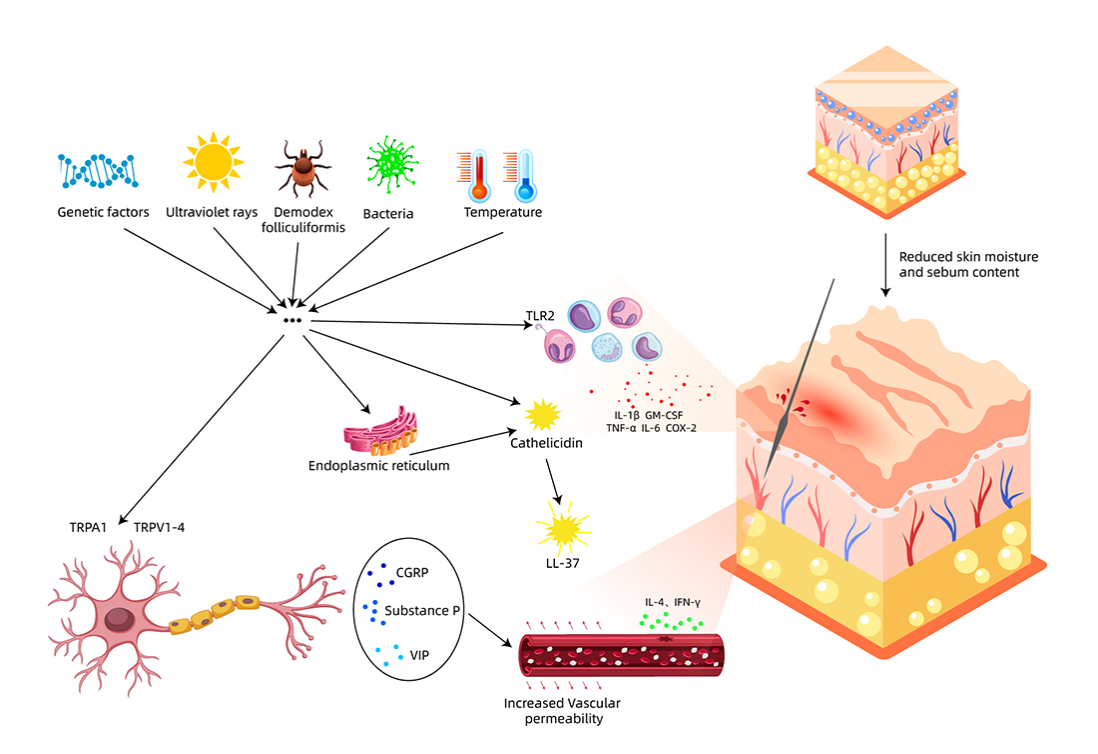
The pathogenesis of rosacea and the underlying principles of collateral puncture therapy.

Ying et al [34] found that collateral puncture therapy can reduce the levels of interleukin-1 β, granulocyte-macrophage colony stimulating factor, tumor necrosis factor α, interleukin-6, and cyclooxygenase-2, while increasing the levels of interleukin-4 and interferon γ. This suggests that collateral puncture therapy enhances the levels of anti-inflammatory cytokines and inhibits the expression of pro-inflammatory cytokines, thereby exerting an anti-inflammatory effect. During treatment, the skin puncturing involved in collateral puncture stimulates the vegetative nervous system, promoting an increase in the secretion of vascular endothelial cells and enhancing the regulatory function of blood vessels. As the collateral puncture disrupts the integrity of blood vessels, it stimulates the function of endothelial cells, leading to the production of a large amount of hormone-like substances that regulate body fluids and blood pressure. Simultaneously, collateral puncture therapy, through physical stimulation causing local capillary rupture, elicits interactive neuroimmune regulation within the microcirculation and also modulates lipid metabolism in the body, reducing the levels of triglycerides, total cholesterol, and other components in the serum [35]. Furthermore, collateral puncture therapy is a simple and cost-effective procedure, with patients reporting immediate relief from symptoms such as facial heat and itching following the treatment. Therefore, this study adopted collateral puncture therapy as the intervention measure (Figure 3).

However, this study is also subject to certain limitations. First, previous studies used IPL treatments that were administered once a month, with 3 to 4 sessions constituting one course of treatment. As an exploratory experiment, our study features a shorter treatment duration and a smaller sample size; therefore, the control group received only 1 treatment session. Future research should aim to address these issues by increasing the sample size and extending the observation period while also increasing the number of treatment sessions for both intervention groups and expanding the follow-up period to 6 months or even 12 months, in order to more objectively assess the efficacy of collateral puncture therapy in treating ETR and its ability to inhibit recurrence. Second, the inclusion and exclusion criteria in this study protocol did not fully control for confounding factors. Subsequent studies should further refine these criteria, including restrictions on the type and frequency of skin care products used by patients, as well as their living environments, among other factors. Furthermore, the evaluation indicators designed for this study primarily focused on patients’ erythema, telangiectasia, and quality of life, without assessing changes in facial skin barrier function. Subsequent studies should incorporate relevant evaluation indicators to gain a deeper understanding of the efficacy of collateral puncture therapy. Lastly, this study did not provide detailed specifications for specific quantitative standards and grading of adverse reactions, nor did it monitor systemic safety indicators. Subsequent studies should refine these aspects by incorporating the assessment of safety indicators before and after treatment, such as routine blood tests and infection status, in order to more accurately compare the occurrence of adverse reactions between the two patient groups.

In summary, collateral puncture therapy for the treatment of ETR possesses a solid theoretical foundation, demonstrates favorable clinical efficacy feedback, is less expensive than photodynamic therapy, and exhibits good safety. However, sufficient clinical evidence to support its use is lacking. The results of this study will provide new insights into the effects and mechanisms of collateral puncture therapy for ETR, thereby complementing the therapeutic options for rosacea, standardizing treatment protocols, potentially reducing disease burden and economic costs, and contributing to both the academic community and the public.

## Data Availability

The datasets generated or analyzed during this study are available from the corresponding author on reasonable request.

## Appendix

Multimedia Appendix 1 SPIRIT (Standard Protocol Items: Recommendations for Interventional Trials) checklist.

Multimedia Appendix 2 Informed consent form.

Multimedia Appendix 3 Outcome forms.

## Abbreviations

IPL: intense pulsed light
SPIRIT: Standard Protocol Items: Recommendations for Interventional Trials
