# Supplementary material for: Treatment of Erythematotelangiectatic Rosacea With Collateral Puncture Therapy: Protocol for a Randomized Controlled Trial
Source: JMIR Res Protoc. 2025 Jun 17;14:e59682. doi: 10.2196/59682 (PMC12214693; doi:10.2196/59682)
Supplement: Multimedia Appendix 2 [file resprot_v14i1e59682_app2.docx]

**Informed Consent Form for the Clinical Study of Treating Erythematotelangiectatic Rosacea with Bloodletting Therapy**

**Ⅰ. Research Background**

Rosacea is a common disfiguring dermatological condition that severely affects the physical and mental health of patients. Currently, the treatment methods for erythematotelangiectatic rosacea have short-term efficacy, noticeable adverse reactions, and impose a significant economic burden on patients, failing to meet clinical needs. In Traditional Chinese Medicine, the pathogenesis of rosacea is attributed to 'stasis' and 'heat', and the treatment principle is to 'clear heat and remove stasis'. Pricking blood therapy is one of the acupuncture therapies in Traditional Chinese Medicine, which can promote the normal circulation and distribution of Qi and blood in the body. It has been used in the treatment of erythematotelangiectatic rosacea, but lacks evidence from evidence-based medicine.

**II. Introduction to the Current Study**

1. The Dermatology Department of Guang'anmen Hospital is a nationally certified clinical research institution. This clinical study is conducted in our department and is led by Associate Chief Physician Yan Yuhe.

2. For this study, the Dermatology Department will provide 4 sessions of pricking bloodletting therapy or 1 session of delicate pulsed light therapy free of charge. A total of 60 rosacea patients will be enrolled.

**The following three criteria are the inclusion standards**

1. Meets the diagnostic criteria for rosacea as defined in the 'Guidelines for the diagnosis and treatment of rosacea in China (2021 edition)', specifically the erythematotelangiectatic type;
2. Aged between 18 and 70 years old (inclusive);
3. Signed an informed consent form.

**The following seven categories of individuals are not suitable for participation in the study**

1. Those with any systemic or active skin disease on the face that could affect the evaluation of the study results (such as connective tissue disease), or those with scars, tattoos, birthmarks, or other pigmentary skin diseases on the affected area that could affect the assessment of skin lesions.
2. Patients with serious primary diseases of the heart, cerebral vessels, liver, kidney, or ematopoietic system, those with mental illnesses or a history of autoimmune diseases; pregnant or breastfeeding women; patients with photosensitivity; individuals with a fear of needles.
3. Patients who have used oral immunomodulators (such as hydroxychloroquine sulfate), β-adrenergic receptor inhibitors (such as carvedilol), and anti-anxiety drugs (such as mirtazapine and paroxetine) for rosacea treatment within 30 days before treatment.
4. Patients who have applied vasoconstrictor drugs on the face (such as 0.5% bromonidine tartrate gel, 1% hydroxymetazoline hydrochloride cream) within 7 days before treatment, as well as those who have used calcineurin inhibitors, antimicrobials, and steroid creams, or have undergone fire-needle therapy, bloodletting, or LED light treatment on the face.
5. Patients who have undergone facial injections, laser treatments, or chemical peels within 30 days before treatment.
6. Those who are currently participating in, or have participated in, another clinical trial within the last 3 months.
7. Patients who cannot tolerate any procedures involved in the trial.

3. In this study, 60 patients will be randomly divided into a control group and a treatment group, with 30 people in each group. Treatment group: Facial pricking bloodletting, once a week for 4 consecutive weeks, a total of 4 treatments; Control group: One session of delicate pulsed light therapy upon enrollment, a total of one treatment. Those who recover during the course may stop the treatment and be included in the efficacy statistics. A follow-up will be conducted 4 weeks after recovery or at the end of the treatment course to observe relapse situations.

If you participate in this clinical study, you will be randomly assigned to one of the two groups mentioned above. The evaluator will not know the treatment method used by the patient until the end of the clinical study and the unblinding.

During the study, researchers will observe and record the patients' subjective symptoms and objective indicators, grade the clinical symptoms, and patients are required to fill out the Clinician’s Erythema Assessment (CEA), Vascular dilation score, Investigator′s Global Assessment (IGA), Patient’s Self-Assessment (PSA), Flushing Symptom Questionnaire (FSQ), Dermatology Life Quality Index (DLQI), and Rosacea-Specific Quality-Of-Life Instrument (RosaQoL). These will be used as evaluation points before treatment, immediately after treatment, and at follow-up.

During the clinical trial, both pricking bloodletting and delicate pulsed light therapy will be provided free of charge.

4. If serious adverse reactions occur during the trial, if poor, ineffective, or clinically valueless treatment effects are discovered during the study process, if major errors in the clinical trial protocol are found making it difficult to evaluate the efficacy, or if there is a significant deviation from a well-designed plan during implementation, making it challenging to assess the treatment effect, the trial may be terminated.

5. During the study, patients' rosacea may improve, but adverse reactions such as redness, itching, and swelling may also occur. If any of these adverse reactions appear, please contact the researchers promptly. If the adverse reaction is mild, the researcher may stop the medication; if the adverse reaction is severe, the researcher will stop the treatment and provide anti-inflammatory and other symptomatic supportive treatments, with the related costs borne by our hospital. For damages related to the study, the Dermatology Department of Guang'anmen Hospital will be responsible for compensation.

6. The treatment provided in this study is not the only treatment option. Other alternative treatments include topical α-adrenergic agonists, which can reduce persistent erythema in the central face but are ineffective for dilated capillaries and inflammatory lesions. It is currently believed that the improvement in erythema by this medication may only be a temporary suppression. Adverse reactions include worsening of erythema or flushing, itching, and skin irritation.

7. Clinical researchers will protect the privacy of patients. At no time will patients' names appear in public publications. However, monitors, auditors, ethics committees, and regulatory authorities should be allowed, within the scope permitted by applicable laws and regulations and without violating the privacy of the subjects, to directly access the subjects' original medical records for verification of the clinical trial procedures and/or data.

8. Patients have the right to choose not to participate in this study and can withdraw at any time without affecting their normal treatment. This trial adheres to the principle of voluntary participation, allowing participants to withdraw at any stage without facing discrimination or retaliation, and their medical treatment and rights will not be affected. However, it is hoped that patients will complete this study unless there are special circumstances. If there are special reasons to withdraw from the study, participants can inform the researchers at any time. If information that may affect the continuation of the trial for the subjects is obtained, the patients or their legal representatives will be promptly informed.

9. If patients have any questions during the clinical study, they can consult the Dermatology Department at 88001870. For complaints or dissatisfaction, contact with the Ethics Committee can be made. The contact number for the Ethics Committee is 88001552.

**Patient Statement**

I have read the subject's informed consent form and fully understand its contents. I am aware that during the clinical trial, if I have reasons such as personal circumstances that prevent me from continuing to participate in the trial, I can refuse to continue or freely withdraw from the trial at any time without any loss of treatment or other benefits. If I have any questions about the clinical trial, I can consult the respective authorities or the Dermatology Department of Guang'anmen Hospital at any time; for research purposes, my medical records are only accessible within the scope of the study. Given the above, I voluntarily agree to participate in this clinical study.

Patient's Signature: Contact Number: Date of Signature:

**Researcher Statement**

I have dutifully fulfilled the obligation of informed consent, explaining to the subject the detailed aspects of the trial, including their rights and the potential benefits and risks.

Research Physician's Signature: Contact Number: Date of Signature:

**刺络法治疗红斑毛细血管扩张型玫瑰痤疮的临床研究**

**知 情 同 意 书**

**一、研究背景：**

玫瑰痤疮是皮肤科常见的损容性皮肤病，严重影响患者身心健康。目前针对红斑毛细血管扩张型玫瑰痤疮的治疗方法，疗效维持时间短、不良反应明显，患者经济负担较重，不能满足临床需求。玫瑰痤疮的中医病因病机为“瘀”“热”，治疗上应“清热祛瘀”。刺络疗法是中医的针灸疗法之一，能够促进体内气血的正常运行与输布，目前已应用在红斑毛细血管扩张型玫瑰痤疮的治疗中，但缺乏循证医学证据。

**二、本次研究的情况介绍：**

1.广安门医院皮肤科是国家认证的临床研究机构，本次临床研究在我院皮肤科开展，由闫雨荷副主任医师主持。

2.此次研究皮肤科会免费提供您4次刺络放血治疗或1次强脉冲光治疗。共需要纳入60例玫瑰痤疮患者。

**以下三类为入选标准：**

（1）符合《中国玫瑰痤疮诊疗指南（2021版）》中玫瑰痤疮的诊断标准，且属于红斑毛细血管扩张型；

（2）年龄18至70岁（包括18和70岁）；

（3）签署知情同意书。

**以下七类不宜参加研究：**

（1）面部患有任何可能影响实验结果评价的全身性或活动性的其他皮肤疾病（如结缔组织病）等，或在患处有瘢痕、纹身、胎记或其他色素性皮肤病等会影响对皮肤病变的评价的患者；

（2）合并了心、脑血管、肝、肾或造血系统等严重的原发性疾病的患者，患有精神疾病或既往自身免疫疾病史的患者；妊娠、哺乳期妇女；光过敏患者；晕针患者；

（3）治疗前30天内应用过，口服免疫调节剂（硫酸羟氯喹片等）、β肾上腺素能受体抑制剂（卡维地洛等）及抗焦虑类药物（米氮平和帕罗西汀等）玫瑰痤疮相关治疗药物的患者；

（4）治疗前7天内面部外用过缩血管药物（0.5%酒石酸溴莫尼定凝胶、1％盐酸羟甲唑啉乳膏等），钙调磷酸酶抑制剂，抗菌剂及激素类药膏，面部接受火针和（或）放血和（或）LED光治疗的患者；

（5）治疗前30天内面部行注射、激光治疗及化学剥脱治疗的患者；

（6）正在参加或最近3个月内参加过其他临床试验；

（7）对试验中任何处置不能耐受的患者。

3.本研究会将60例患者随机分为对照组与治疗组，每组30人。治疗组：面部刺络放血，每周刺络1次，连续4周，共治疗4次；对照组：入组后给予治疗强脉冲光1次，共治疗1次。中途痊愈者可停止治疗，纳入疗效统计。痊愈后或疗程结束后4周随访1次，观察复发情况。

如参加本临床研究，将随机分入以上两组，评价者不知道患者所使用的治疗方法，直至临床研究全部结束揭盲之前。

研究期间，研究者观察并记录患者的主观症状和客观指标，对临床症状进行分级评分，患者需填写持续性红斑医生评估量表（CEA）、毛细血管扩张医生评分量表、医生整体评价法（IGA）、患者自身评价量表（PSA）、阵发性潮红量表（FSQ）、皮肤病生活质量量表（DLQI）和玫瑰痤疮生活质量量表（RosaQoL），分别选择治疗前，治疗结束即刻，随访时作为评价时点。

临床试验期间，刺络放血以及强脉冲光治疗均免费。

4.若在试验中发生严重不良反应、研究过程中发现治疗效果不好、甚至无效、不具有临床价值者；在试验中发现所定临床试验方案有重大失误、难以评价疗效；或对一项设计较好的方案，在实施中发生重大偏差，难以评价治疗效应时，试验可能被终止。

5.在研究过程中，患者的玫瑰痤疮可能会减轻，也可能出现发红、瘙痒、水肿等不良反应，若出现上述不良反应请及时与研究者联系。若不良反应轻，研究者可予以停止用药；若不良反应较重，研究者会停止治疗并予以抗炎等对症支持治疗，相关费用将由本医院承担。发生研究相关损害，由广安门医院皮肤科负责赔偿。

6.本研究所给予的治疗方案并非唯一的治疗方案，其他可替代的治疗有：外用α肾上腺受体激动剂，外用α肾上腺受体激动剂能减少面中部的持久性红斑，但对已扩张的毛细血管及炎性皮损无效。目前认为该药对红斑的改善可能只是暂时性抑制。不良反应包括红斑或潮红加重、瘙痒和皮肤刺激等。

7.临床研究者会保护患者的隐私权，无论何时，患者的姓名均不会出现在公开的刊物上，但监查员、稽查员、伦理委员会和管理当局应被准予在不违反适用法律和法规所准许的范围内，在不侵犯受试者的隐私的情况下，直接查阅受试者的原始医疗记录以便核查临床试验的程序和/或数据。

8.患者有权不选择参与本次研究，患者也有权随时退出，并不影响其正常治疗。本试验秉承自愿参与研究的原则，在试验的任何阶段有随时退出研究并且不会遭到歧视或报复，其医疗待遇与权益不受影响的权力，但希望患者在无特殊情况下一定完成本次研究。如有特殊原因需要退出研究，可随时告知研究者。如果得到可能影响受试者继续参加试验的信息，患者或其合法代理人将及时得到通报。

9.如果在临床研究期间患者有疑问可以咨询皮肤科，电话为：88001870。若有不满、抱怨，可与伦理委员会联系。伦理委员会联系电话为88001552。

**患者声明**

本人已参阅了受试者知情同意书，对其内容已充分理解。本人详知在临床试验期间，若因个人情况等出现不能继续参与试验的理由时，随时可以中途拒绝持续参与试验或自由退出试验过程，亦不会因此而受到诊疗及其他任何利益上的损失。本人对临床试验产生疑问时，可以随时向各机关主管研究人员或广安门医院皮肤科咨询；从研究目的出发，对本人的医疗记录仅限于研究范围内可以直接阅览。介于上述情况，本人自愿同意参与本临床研究。

患者签字： 联系电话 签字日期：

**研究者声明**

已经认真履行了知情告知义务，向受试者解释了试验的详细情况，包括其权利以及可能的受益和风险。

研究医师签字： 联系电话： 签字日期：
