# Supplementary material for: Treatment of Erythematotelangiectatic Rosacea With Collateral Puncture Therapy: Protocol for a Randomized Controlled Trial
Source: JMIR Res Protoc. 2025 Jun 17;14:e59682. doi: 10.2196/59682 (PMC12214693; doi:10.2196/59682)
Supplement: Multimedia Appendix 3 [file resprot_v14i1e59682_app3.docx]

| **Clinician’s Erythema Assessment, CEA** | | | | | |
| --- | --- | --- | --- | --- | --- |
| 0 | 1 | 2 | 3 | 4 | Score |
| Clear skin with no signs of erythema | Almost clear;  slight redness | Mild erythema, definite redness | Moderate erythema; marked redness | Severe erythema; fiery redness |  |

| **Vascular dilation score** | | | | | |
| --- | --- | --- | --- | --- | --- |
| 0 | 1 | 2 | 3 | 4 | Score |
| No lesions | Mild: A small amount of small blood vessels with a diameter of<0.2mm and an area of<5% of the face | Moderate: There are many small blood vessels and/or a small number of large blood vessels with a diameter>0.2mm, covering an area of 5% to 15% of the face | Severe: There are many small blood vessels and/or a small number of large blood vessels with a diameter greater than 0.2mm, covering an area of 15% to 30% of the face | Very serious: a large number of small and/or large blood vessels, with an area greater than 30% of the face. |  |

| **Investigator′s Global Assessment, IGA** | | | | | | |
| --- | --- | --- | --- | --- | --- | --- |
| -1 | 0 | 1 | 2 | 3 | 4 | Score |
| aggravation | No improvement  (<25% clearance) | Mild improvement  (25%-49% clearance) | Moderate improvement  (50%-74% clearance) | Significant improvement  (75%-99% clearance) | Complete improvement |  |

| **Patient′s Self-Assessment, PSA** | | | | | | |
| --- | --- | --- | --- | --- | --- | --- |
| -1 | 0 | 1 | 2 | 3 | 4 | Score |
| aggravation | No improvement  (<25% clearance) | Mild improvement  (25%-49% clearance) | Moderate improvement  (50%-74% clearance) | Significant improvement  (75%-99% clearance) | Complete improvement |  |

**Flushing Symptom Questionnaire（FSQ）**

Regarding the following questions, please consider the possible redness symptoms you may have experienced in the past 24 hours (including skin redness, fever, stinging, or itching)

1. How many times have you experienced flushing symptoms in the past 24 hours (including skin redness, fever, stinging, or itching reactions with options 1, 2, 3, or more)
2. Overall, how did you evaluate your flushing symptoms in the past 24 hours? (including skin redness, fever, stinging or itching)

No mild moderate severe extremely serious


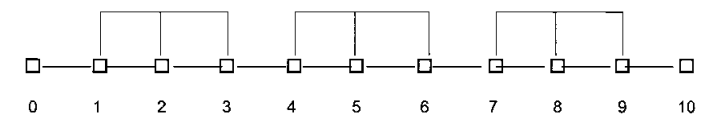


1. How long has your longest flushing symptom lasted in the past 24 hours (including skin redness, fever, stinging or itching)?(No,<5 minutes, 5 minutes to 2 hours in increments of 5 minutes,>2 hours, unknown)
2. Overall, how annoying have your flushing symptoms been in the past 24 hours? (including skin redness, fever, stinging or itching)

Not annoying a bit annoying annoying very annoying extremely annoying


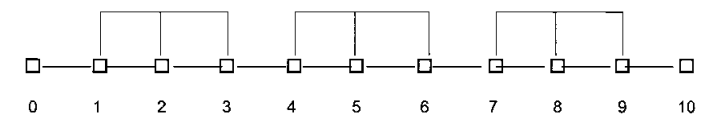


1. How do you evaluate skin redness caused by redness in the past 24 hours?

No mild moderate severe extremely serious


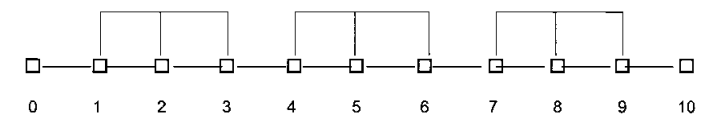


1. How do you evaluate skin fever caused by redness in the past 24 hours?

No mild moderate severe extremely serious


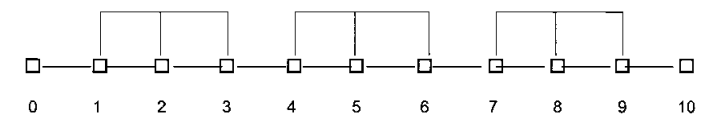


1. How do you evaluate skin irritation caused by redness in the past 24 hours?

No mild moderate severe extremely serious


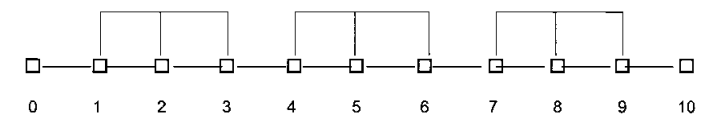


1. How do you evaluate skin itching caused by redness in the past 24 hours?

No mild moderate severe extremely serious


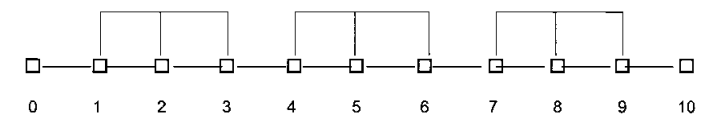


The next question refers to the difficulty sleeping (falling asleep or maintaining sleep) caused by the flushing symptoms last night

1. How annoying was it last night that I had difficulty falling asleep due to flushing?

Not annoying a bit annoying annoying very annoying extremely annoying


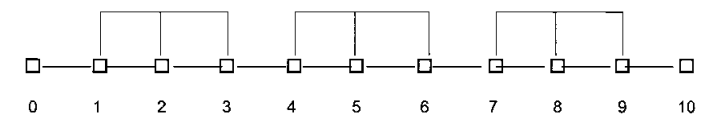


| **Rosacea-Specific Quality-Of-Life Instrument, RosaQoL** | |
| --- | --- |
| Questions (All about your situation in the past week) | Severity |
| 1. I worry that my rosacea may be serious | □ Never □ Rarely □ Sometimes  □ Often □ Always |
| 2. My rosacea burns or stings | □ Never □ Rarely □ Sometimes  □ Often □ Always |
| 3. I worry about getting scars from my rosacea | □ Never □ Rarely □ Sometimes  □ Often □ Always |
| 4. I worry that my rosacea may get worse | □ Never □ Rarely □ Sometimes  □ Often □ Always |
| 5. I worry about side effects from rosacea medications | □ Never □ Rarely □ Sometimes  □ Often □ Always |
| 6. My rosacea is irritated | □ Never □ Rarely □ Sometimes  □ Often □ Always |
| 7. I am embarrassed by my rosacea | □ Never □ Rarely □ Sometimes  □ Often □ Always |
| 8. I am frustrated by my rosacea | □ Never □ Rarely □ Sometimes  □ Often □ Always |
| 9. My rosacea makes my skin sensitive | □ Never □ Rarely □ Sometimes  □ Often □ Always |
| 10. I am annoyed by my rosacea | □ Never □ Rarely □ Sometimes  □ Often □ Always |
| 11. I am bothered by the appearance of my skin (redness, blotchiness) | □ Never □ Rarely □ Sometimes  □ Often □ Always |
| 12. My rosacea makes me feel self-conscious | □ Never □ Rarely □ Sometimes  □ Often □ Always |
| 13. I try to cover up my rosacea (with makeup) | □ Never □ Rarely □ Sometimes  □ Often □ Always |
| 14. I am bothered by persistence/reoccurrence of my rosacea | □ Never □ Rarely □ Sometimes  □ Often □ Always |
| 15. I avoid certain foods or drinks because of my rosacea | □ Never □ Rarely □ Sometimes  □ Often □ Always |
| 16. My skin feels bumpy (uneven, not smooth, irregular) | □ Never □ Rarely □ Sometimes  □ Often □ Always |
| 17. My skin flushes | □ Never □ Rarely □ Sometimes  □ Often □ Always |
| 18. My skin gets irritated easily (cosmetics, aftershaves, cleansers) | □ Never □ Rarely □ Sometimes  □ Often □ Always |
| 19. My eyes bother me (feel dry or gritty) | □ Never □ Rarely □ Sometimes  □ Often □ Always |
| **score：**\|___\|___\| | |

Note: Never, rarely, sometimes, often, and always correspond to 0, 1, 2, 3, and 4 points respectively, with a maximum score of 76 points.

| **Dermatology Life Quality Index, DLQI** | |
| --- | --- |
| Questions | Severity |
| 1. Over the last week, how itchy, sore, painful or stinging has your skin been? | □ Not at all □ A little  □ A lot □ Very much |
| 1. Over the last week, how embarrassed or self conscious have you been because of your skin? | □ Not at all □ A little  □ A lot □ Very much |
| 1. Over the last week, how much has your skin interfered with you goingshopping or looking after your home or garden? | □ Not at all □ A little  □ A lot □ Very much |
| 1. Over the last week, how much has your skin influenced the clothes you wear? | □ Not at all □ A little  □ A lot □ Very much |
| 1. Over the last week, how much has your skin affected any social or leisure activities? | □ Not at all □ A little  □ A lot □ Very much |
| 1. Over the last week, how much has your skin made it difficult for you to do any sport? | □ Not at all □ A little  □ A lot □ Very much |
| 1. Over the last week, has your skin prevented you from working or studying? | □ Not at all □ A little  □ A lot □ Very much |
| 1. Over the last week, how much has your skin created problems with your partner or any of your close friends or relatives? | □ Not at all □ A little  □ A lot □ Very much |
| 1. Over the last week, how much has your skin caused any sexual difficulties? | □ Not at all □ A little  □ A lot □ Very much |
| 1. Over the last week, how much of a problem has the treatment for your skin been. for example by making your home messy, or by taking up time? | □ Not at all □ A little  □ A lot □ Very much |
| **score：**\|___\|___\| | |

Note: Not at all, A little, A lot and Very much correspond to 0, 1, 2, and 3 points respectively, with a maximum score of 30 points.

| **持续性红斑医生评估量表(Clinician’s Erythema Assessment, CEA)** | | | | | |
| --- | --- | --- | --- | --- | --- |
| 0分 | 1分 | 2分 | 3分 | 4分 | 分数 |
| 无 | 几乎无红斑；  轻微发红 | 轻度红斑；  明显发红 | 中度红斑；  显著发红 | 重度红斑；  鲜红 |  |

| **毛细血管扩张医生评估量表** | | | | | |
| --- | --- | --- | --- | --- | --- |
| 0分 | 1分 | 2分 | 3分 | 4分 | 分数 |
| 无 | 轻度：少量直径<0.2mm的小血管，面积<面部5% | 中度：较多小血管和（或）少量直径>0.2mm的大血管，面积为面部5%~15% | 重度：较多小血管和（或）少量直径>0.2mm的大血管，面积为面部15%~30% | 非常严重：大量小血管和（或）大血管，面积＞面部 30%。 |  |

| **医生整体评价法（Investigator′s Global Assessment, IGA）** | | | | | | |
| --- | --- | --- | --- | --- | --- | --- |
| -1分 | 0分 | 1分 | 2分 | 3分 | 4分 | 分数 |
| 加重 | 无改善  （< 25%清除） | 轻度改善  （25%-49%清除） | 中度改善  （50%-74%清除） | 显著改善  （75%-99%清除） | 完全改善 |  |

| **患者自身评价量表（Patient′s Self-Assessment, PSA）** | | | | | | |
| --- | --- | --- | --- | --- | --- | --- |
| -1分 | 0分 | 1分 | 2分 | 3分 | 4分 | 分数 |
| 加重 | 无改善  （< 25%清除） | 轻度改善  （25%-49%清除） | 中度改善  （50%-74%清除） | 显著改善  （75%-99%清除） | 完全改善 |  |

**阵发性潮红量表（FSQ）**

关于以下问题，请思考您在过去24小时内可能出现的潮红症状（包括皮肤发红、发热、刺痛或发痒）

2.在过去24小时内，您出现过多少次潮红症状（包括皮肤发红、发热、刺痛或瘙痒反应选项没有、1、2、3或更多）

3.**总体感觉**，在过去的24小时内，您如何评价您的潮红症状？（包括皮肤发红、发热、刺痛或瘙痒）

没有 轻微 中等程度 严重 极严重


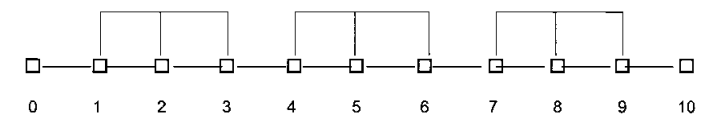


4.在过去的24小时内，您最长的潮红症状持续了多长时间（包括皮肤发红、发热、刺痛或发痒）？（没有、＜5分钟，5分钟到2个小时，以5分钟为增量，＞2个小时，不知道）

5.**总体感觉，**在过去24小时内，你的潮红症状有多**烦人**？（包括皮肤发红、发热、刺痛或瘙痒）

不烦人 有点烦人 烦人 非常烦人 极烦人


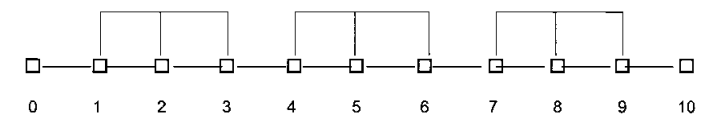


6.在过去的24小时内，你如何评价因潮红而导致的**皮肤发红**？

没有 轻微 中等程度 严重 极严重


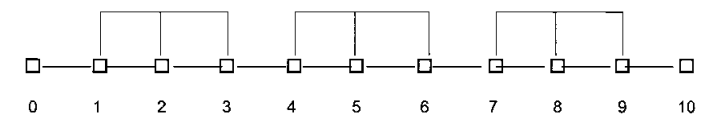


7.在过去的24小时内，你如何评价因潮红而导致的**皮肤发热**？

没有 轻微 中等程度 严重 极严重


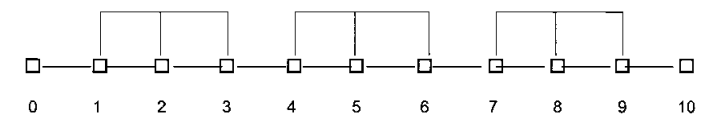


8.在过去的24小时内，你如何评价因潮红而导致的**皮肤刺痛**？

没有 轻微 中等程度 严重 极严重


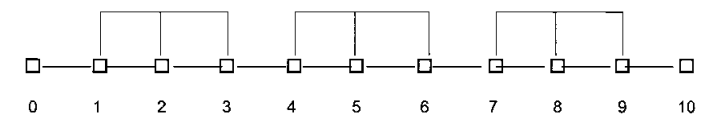


9.在过去的24小时内，你如何评价因潮红而导致的**皮肤瘙痒**？

没有 轻微 中等程度 严重 极严重


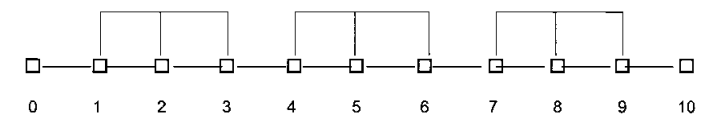


接下来的问题是指昨晚由于潮红症状导致的睡眠困难（入睡或保持睡眠）

10.脸红症状导致你昨晚睡眠困难吗？回答：是，否，如果否，则答题结束。

11.昨天晚上由于潮红而难以入睡是多么**烦人**的事呢？

不烦人 有点烦人 烦人 非常烦人 极烦人


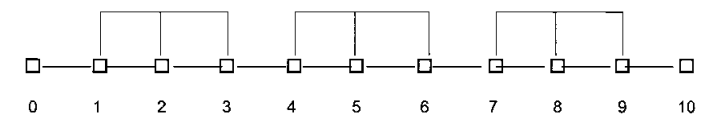


| **玫瑰痤疮生活质量量表Rosacea-Specific Quality-Of-Life Instrument（RosaQoL）** | |
| --- | --- |
| 问题（注意以下问题均为**过去一周**内您的情况） | 严重程度 |
| 1. 我担心玫瑰痤疮很严重 | □从不 □很少 □有时 □经常 □总是 |
| 2. 玫瑰痤疮发作我的脸会发热或刺痛 | □从不 □很少 □有时 □经常 □总是 |
| 3. 我担心玫瑰痤疮会留下疤痕 | □从不 □很少 □有时 □经常 □总是 |
| 4. 我担心我的玫瑰痤疮会加重 | □从不 □很少 □有时 □经常 □总是 |
| 5. 我担心玫瑰痤疮的药物治疗会有副作用 | □从不 □很少 □有时 □经常 □总是 |
| 6. 我的玫瑰痤疮易被刺激发作 | □从不 □很少 □有时 □经常 □总是 |
| 7. 我因为玫瑰痤疮感到尴尬 | □从不 □很少 □有时 □经常 □总是 |
| 8. 我因为玫瑰痤疮感到挫败 | □从不 □很少 □有时 □经常 □总是 |
| 9. 玫瑰痤疮使我的皮肤敏感 | □从不 □很少 □有时 □经常 □总是 |
| 10. 我常因玫瑰痤疮生气 | □从不 □很少 □有时 □经常 □总是 |
| 11. 我为我的皮肤感到困扰（发红、斑点） | □从不 □很少 □有时 □经常 □总是 |
| 12. 我总是感觉到我有玫瑰痤疮 | □从不 □很少 □有时 □经常 □总是 |
| 13. 玫瑰痤疮一直不消退或者反复发作很困扰我 | □从不 □很少 □有时 □经常 □总是 |
| 14. 因为玫瑰痤疮我不吃某些事物或饮品 | □从不 □很少 □有时 □经常 □总是 |
| 15. 我的皮肤很粗糙（不平、不光滑） | □从不 □很少 □有时 □经常 □总是 |
| 16. 我的皮肤时常潮红 | □从不 □很少 □有时 □经常 □总是 |
| 17. 我的皮肤易受到刺激（化妆品、爽肤水和洗面奶） | □从不 □很少 □有时 □经常 □总是 |
| 18. 我时常想到我的玫瑰痤疮 | □从不 □很少 □有时 □经常 □总是 |
| 19. 因为玫瑰痤疮我会避免接触一些环境因素（炎热、湿度大、寒冷） | □从不 □很少 □有时 □经常 □总是 |
| **RosaQoL总评分：**\|___\|___\|分 | |

注：从不、很少、有时、经常、总是，分别对应0、1、2、3、4分，满分为76分。

| **皮肤病生活质量量表Dermatology Life Quality Index (DLQI)** | |
| --- | --- |
| 问题 | 严重程度 |
| 1、过去一周内，您感到皮肤痒、疼痛、触痛、刺痛了吗？ | □无 □少些 □严重 □非常严重 |
| 2、过去一周内，您的皮肤问题使您感到尴尬或自卑程度如何？ | □无 □少些 □严重 □非常严重 |
| 3、过去一周内，皮肤问题对您的购物、做家务、整理庭院影响如何？ | □无 □少些 □严重 □非常严重 |
| 4、过去一周内，皮肤问题对您穿衣服影响程度如何？ | □无 □少些 □严重 □非常严重 |
| 5、过去一周内，皮肤问题对您的社交或休闲生活的影响有多大？ | □无 □少些 □严重 □非常严重 |
| 6、过去一周内，皮肤问题对您运动影响有多大？ | □无 □少些 □严重 □非常严重 |
| 7、过去一周内，您的皮肤问题对学习或工作影响有多大？ | □无 □少些 □严重 □非常严重 |
| 8、过去一周内，皮肤问题妨碍了您和爱人、亲戚、亲密的朋友间的交往了吗？ | □无 □少些 □严重 □非常严重 |
| 9、过去一周内，皮肤问题给您的性生活造成了影响有多大？ | □无 □少些 □严重 □非常严重 |
| 10、过去一周内，由于治疗您的皮肤毛病给您造成了麻烦有多少？如占用了您很多时间或把家里弄得一团糟？ | □无 □少些 □严重 □非常严重 |
| **DLQI总评分：**\|___\|___\|分 | |

注：无、少些、严重、非常严重，分别对应0、1、2、3分，满分为30分。
